# Pre‐service teachers' explicit and implicit stereotypes towards pupils with different special educational needs

**DOI:** 10.1111/bjep.70041

**Published:** 2025-10-07

**Authors:** Charlotte S. Schell, Hannah Kleen, Charlotte Dignath, Nathalie John, Mareike Kunter

**Affiliations:** ^1^ DIPF | Leibniz Institute for Research and Information in Education Frankfurt am Main Germany; ^2^ IDeA Center for Research on Individual Development and Adaptive Education of Children at Risk Frankfurt am Main Germany; ^3^ Institute of Psychology Goethe University Frankfurt Frankfurt am Main Germany

**Keywords:** autism, direct methods, Down syndrome, dyslexia, inclusion, indirect methods, stereotypes

## Abstract

**Background:**

Successful inclusion in education depends heavily on the attitudes of teachers, and stereotypes play a significant role in shaping these attitudes. However, social desirability bias may limit direct measures of stereotypes. Combining direct and indirect measures offers better insights. But studies on SEN‐specific stereotypes combining these measures are rare.

**Aims:**

This study aims to investigate and compare pre‐service teachers' explicit and implicit stereotypes towards autistic pupils, pupils with Down syndrome, and pupils with dyslexia using direct and indirect measures.

**Sample:**

Our sample consisted of *N* = 76 German pre‐service teachers with an average age of 22.75 years (*SD* = 3.32), of which 61% identified as female.

**Methods:**

We assessed explicit stereotypes via a questionnaire and implicit stereotypes using lexical decision tasks. To compare explicit and implicit stereotypes, we computed Kendall's tau correlation coefficients.

**Results:**

Participants rated stereotypical adjectives significantly above the neutral midpoint for all three groups. They responded significantly faster to stereotypical than to non‐stereotypical words in the lexical decision tasks, with large effect sizes for explicit ratings and medium effect sizes for implicit measures. Explicit and implicit stereotypes did not correlate significantly with one another.

**Conclusion:**

The study found that pre‐service teachers clearly endorsed explicit stereotypes and showed implicit associations in line with these stereotypes, especially towards autistic pupils and those with Down syndrome, while patterns for dyslexia were less pronounced. These results underline how common such stereotypes are in educational contexts and suggest that interventions should address both explicit and implicit bias.

## INTRODUCTION

Over the last decades, inclusion has taken centre stage within the educational context (United Nations' Convention on the Rights of Persons with Disabilities, 2006). Concerning if and how teachers implement inclusive practices, attitudes towards inclusion in general as well as towards pupils with special educational needs (SEN) particularly impact their behaviour, and more specifically, their teaching methods (Avramidis & Norwich, 2002; Yeo et al., 2016).

Given the importance of (pre‐service) teachers' attitudes, several studies and meta‐analyses focus on global attitudes towards inclusion (Avramidis & Norwich, 2002; Charitaki et al., 2024; Dignath et al., 2022; Guillemot et al., 2022). However, it is important to look at the topic in a more nuanced way: First, the group of students with SEN is notably heterogeneous (Delaney, 2016; Lindsay, 2007) and can therefore activate different attitudes among (pre‐service) teachers. In recent years, some studies focused more specifically on attitudes towards certain groups. Derguy et al. (2023), for example, investigated attitudes, or more precisely, an affective component of attitudes, towards autistic people. Other studies (Draaisma, 2009) have investigated stereotypes as a cognitive component of attitudes or even explored implicit stereotypes (Enea‐Drapeau et al., 2012) towards autistic people and people with Down syndrome, respectively. Second, attitudes are a generic category and are often divided into affective, cognitive, and behavioural components (Eagly & Chaiken, 1993). Additionally, said components can be explicit, meaning controlled and conscious, or implicit, meaning automatic and unconscious (Fazio, 1995).

### Stereotypes and their significance in the context of inclusion

Following the multicomponent model of attitudes (Eagly & Chaiken, 1993), there are three components of attitudes: the affective, the cognitive and the behavioural component. The behavioural component refers to behavioural tendencies towards an object, an individual or a group (Eagly & Chaiken, 1993). The affective component involves emotions or feelings directed towards an object, an individual or a group (Eagly & Chaiken, 1993). The cognitive component consists of knowledge, beliefs and expectations about social groups and their behaviours (Eagly & Chaiken, 1993).

Stereotypes belong to the cognitive component and refer to generalized representations regarding the characteristics, and behaviours of individuals belonging to a specific social group (Sherman, 1996). Individual differences between group members are not considered (Allport, 1954). In this context, stereotypes must be differentiated from personal beliefs: While stereotypes are shared representations within a culture (McGarty et al., 2002), personal beliefs refer to a personal conviction that is accepted as true and therefore might differ between persons. Thus, a person's stereotypes may or may not align with their personal beliefs (Devine, 1989). However, even if people may not agree with certain stereotypes, they can still impact their information processing and subsequently behaviour. This is due to social categorization processes which help categorize and simplify the world around us. Because we have limited cognitive capacity, stereotypes can reduce the complexity of the social environment to lower the cognitive load and save energy (Allport, 1954; Tajfel et al., 1971). Dual process models (Fazio, 1995) explain how cognitive capacity as well as motivation and opportunity influence the attitude–behaviour link. The relevance of stereotypes becomes clear here, as they can influence people's behaviour, both through a more controlled path and through a more automatic path. It illustrates that both implicit and explicit stereotypes of (pre‐service) teachers could play significant roles. Controlled and consciously accessible stereotypes are labelled as explicit stereotypes and generally require more cognitive capacity, more motivation and opportunity compared with implicit stereotypes. These are automatic and not directly accessible and therefore less reliant on the before‐mentioned resources (Greenwald & Banaji, 1995; Nosek et al., 2007). Different measures are required to capture these forms of stereotype activation (Nosek et al., 2007). To assess explicit stereotypes, direct measures such as self‐report questionnaires or interviews are used, which give people the opportunity to reflect. Contrastingly, indirect measures do not rely on self‐reports but are inferred through indirect methods like response latency tasks, which allow researchers to detect automatic mental associations that may not be consciously endorsed (De Houwer, 2006; Greenwald & Banaji, 1995).

Recent studies on direct measures and the topic of inclusion have shown the risk of social desirability bias and have advertised against using only direct measures in this context (Lüke & Grosche, 2018a, 2018b). Unsurprisingly, correlations between direct and indirect measures are often low, especially concerning socially sensitive topics such as stereotypes (Hofmann et al., 2005). Furthermore, indirect measures reliably predict behaviour (Hofmann et al., 2005). It thus seems important to consider explicit and implicit measures when investigating stereotypes. In relation to the school context, measuring stereotypes towards pupils with SEN as one group may not be nuanced or contextualized enough, as different SEN evoke different stereotypes (Krischler et al., 2018; Norwich, 2013). Different, more nuanced stereotypes are also taken up in the Stereotype Content Model (Fiske, 2012; Fiske et al., 2002). Among other research areas, it has been influential in educational research on teachers' perceptions of pupils (Krämer & Zimmermann, 2025; Krischler et al., 2018; Krischler & ten Pit‐Cate, 2019) and has been the motivation for other questionnaires exploring stereotypes in the school context (Schell et al., 2024).

### Stereotypes about different groups of pupils with SEN

From the many groups of pupils with SEN we focused on three groups that are prevalent and differ substantially in regard to symptoms, SEN and stereotypes (National Autistic Society, n.d.; American Psychiatric Association, 2013; Bougeard et al., 2021; European Dyslexia Association, 2020; World Health Organization, 2023): Autistic pupils, pupils with Down syndrome, and pupils with dyslexia.^1^ Given the already existing research on these groups, we expect teachers to perceive them in very diverse ways.

Firstly, regarding autism, the stereotypical image of both the general public as well as teachers is largely influenced by the portrayal of autistic persons in the media (Draaisma, 2009; Ressa & Goldstein, 2021). This portrayal is often not representative of the entirety of autism (Nordahl‐Hansen & Øien, 2021), which affects 1 in 65 children in Europe (Bougeard et al., 2021). Symptoms are plenty and include persistent challenges in social communication and interaction, restricted or repetitive patterns of behaviour, and hyper‐ or hyporeactivity to sensory input (American Psychiatric Association, 2013). The UK National Autistic Society points out that, as a spectrum condition, it affects people in diverse ways and individual abilities differ greatly (National Autistic Society, n.d.). However, both the media portrayal as well as the stereotypes often focus on only a few characteristics: the idea of autistic persons being extremely intelligent and/or savants is very common (Draaisma, 2009; Mallipeddi et al., 2024). Furthermore, autistic people are sometimes stereotypically seen as dangerous (Gillespie‐Lynch et al., 2021). When it comes specifically to pre‐ and in‐service teachers and autistic pupils, plenty of studies have examined general knowledge (of diagnostic criteria for example), general or affective attitudes and self‐efficacy expectations (Corona et al., 2017; Lu et al., 2020; Wittwer et al., 2024), but only Schell et al. (2024) explored stereotypes. They found that pre‐service teachers strongly associated autistic pupils with several stereotypical traits. In addition to being intelligent, savants and displaying behavioural problems, they also rated them as very introverted, impulsive, impatient, dependent, uncommunicative, and not socially competent, among others (Schell et al., 2024). As for implicit stereotypes, to the best of our knowledge, there are no studies regarding autism.

Secondly, research on stereotypes regarding Down syndrome, recognized by the presence of an additional 21st chromosome (American Psychiatric Association, 2013), is limited. Worldwide, the number of children, and subsequently pupils with Down syndrome, has been growing and it is the most common chromosomal condition in the world, though in Europe, numbers vary by country (World Health Organization, 2023). The existing research shows that both the general public as well as teachers (Gilmore et al., 2003; Gunn & Cuskelly, 1991) stereotypically describe children with Down syndrome as content, warm, loving, and friendly (Canton et al., 2023; Rodríguez et al., 2018). At the same time, they are also seen as less competent (Canton et al., 2023; Rodríguez et al., 2018), less determined, and easier to distract (Cuskelly & Gunn, 2006; Gunn & Cuskelly, 1991). Among other things, pre‐service teachers rated pupils with Down syndrome as being tolerant, warm‐hearted, sincere, and good‐natured though also impatient, dependent, awkward, displaying behavioural problems, low‐achieving, and even stupid (Schell et al., 2024). As for implicit stereotypes, Enea‐Drapeau et al. (2012) used positive and negative adjectives concerning children with Down syndrome in an Implicit Association Test (IAT), showing a negative implicit bias. But because the categories were positive and negative, the IAT did not measure stereotypes but rather an affective component of attitudes. To our knowledge, no other studies investigated implicit stereotypes in the context of Down syndrome.

Third, dyslexia, a specific learning disability characterized by challenges in reading accuracy, reading fluency, and comprehension (American Psychiatric Association, 2013) with a prevalence of 9–12% (European Dyslexia Association, 2020), is often combined with other learning disorders regarding stereotype research. Studies show that pupils with learning difficulties in general are stereotypically described as incompetent and lower achieving (Krischler et al., 2018), although a learning disorder per se is not related to low intelligence (American Psychiatric Association, 2013). Gibbs and Elliott (2015) show that the labels ‘dyslexia’ and ‘learning difficulties’ have different impacts on teachers' beliefs; therefore, it might be reasonable to look specifically at stereotypes regarding dyslexia. Schell et al. (2024) found that pre‐service teachers stereotypically rated dyslexic pupils similarly with traits, such as lazy, stupid, impatient, dependent, awkward, low‐achieving, uncommunicative, displaying behavioural problems, though also to a lesser degree warm‐hearted. To this day, however, no studies have examined implicit stereotypes regarding dyslexia.

### The present study

In this study, we aim to assess stereotypical traits that pre‐service teachers' explicitly and implicitly associated with the three groups of pupils, using and comparing both direct and indirect measures. We chose pre‐service teachers because teachers' attitudes and their components as well as their beliefs act as filters for further perception and interpretation of events (Fives & Buehl, 2012). Therefore, it seems important to target potentially dysfunctional beliefs at an early stage of the professional career. We postulate the following research questions:

1. Regarding autistic pupils:
  - Which stereotypical traits do pre‐service teachers associate explicitly with autistic pupils?
  - Which stereotypical traits do pre‐service teachers associate implicitly with autistic pupils?
  - Comparing explicit and implicit associations, do they correlate? Based on the literature, we expect no or only weak correlations. We expect the topic in general and especially regarding disadvantaged groups of pupils to be socially sensitive, with motivational influences, and therefore social desirability, possibly playing a role. This means that when asked directly, pre‐service teachers could be motivated and able to control their answer to a certain extent resulting in less stereotypical responses. This should not be the case for our indirect measure.
2. Regarding pupils with Down syndrome:
  - Which stereotypical traits do pre‐service teachers associate explicitly with pupils with Down syndrome?
  - Which stereotypical traits do pre‐service teachers associate implicitly with pupils with Down syndrome?
  - Comparing explicit and implicit associations, do they correlate? As outlined above, we expect no or only weak correlations.
3. Regarding pupils with dyslexia:
  - Which stereotypical traits do pre‐service teachers associate explicitly with pupils with dyslexia?
  - Which stereotypical traits do pre‐service teachers associate implicitly with pupils with dyslexia?
  - Comparing explicit and implicit associations, do they correlate? As outlined above, we expect no or only weak correlations.

## METHOD

### Participants

The sample consisted of *N* = 76 German pre‐service teachers with an average age of 22.75 years (*SD* = 3.32). 61% identified as female, 39% as male, and 0% as non‐binary. They were recruited through seminars and lectures at one German university. While the semester of study ranged from the first semester to the 12th semester, the median semester was three, with one‐fourth of all participants (24%) being in their first semester of study. Regarding the type of teaching degree programme, 13% were qualifying for elementary school, 34% for the academic, 25% for the intermediate, 4% for the vocational, and 24% for the SEN track. This suggests that the sample size was sufficient to detect effects within the expected range.

### Procedure

The study was conducted in a laboratory setting. After the participants gave informed consent, the assessment started with three lexical decision tasks (LDT) in randomized order and with breaks in between. After finishing the LDTs, participants filled out the online questionnaires about their explicit stereotypes and demographic data. The study finished with a debriefing and compensation for participation.

### Measures

#### Direct measures

To assess pre‐service teachers' explicit associations, we used an adapted version (Schell et al., 2024) of a questionnaire by Fiske et al. (2002). The participants rated all adjectives in response to the question ‘What do you believe most people think: How [trait] are pupils with autism [Down syndrome, dyslexia] thought to be?’ on a 6‐point Likert scale ranging from 1 = *not at all* to 6 = *very much*. There were two reasons why we decided to use this wording. Firstly, following Fiske et al. (2002), we wanted to reduce social desirability, as participants would feel less pressure to provide answers that align with perceived social expectations. The second and most important reason was that we were not interested in the personal beliefs of the pre‐service teachers but rather in the cultural stereotypical associations (Fiske et al., 2002). As previously explained, stereotypes are generalized representations usually shared within a culture, and these do not necessarily align with someone's personal beliefs (Devine, 1989).

#### Indirect measures

To assess pre‐service teachers' implicit stereotypes, we used three LDTs: one for each type of special educational need. We chose the LDT as it allowed us to assess stereotype‐based semantic associations without the need for explicit categorization labels or contrastive target groups, which are structurally required in IAT paradigms but not conceptually appropriate for our research focus. Moreover, the LDT enabled us to avoid artificial dichotomies (e.g., ‘autistic’ vs. ‘neurotypical’) and problematic labelling that would have been necessary in implicit association tests.

This method is based on the comparison of response times to lexical stimuli that are presented after a prime. This response time is a measure of the accessibility of a lexical stimulus in the mind of the participant (Libben, 2008). A stereotypical word as a lexical stimulus should be more accessible than a non‐stereotypical one and therefore, the response time should be shorter (Chasteen et al., 2002; Marx & Ko, 2012; Wittenbrink et al., 1997). Aside from stereotypical and non‐stereotypical words, non‐words are part of the LDT to prevent participants from responding mindlessly and to allow for task‐accuracy checks. Participants are usually told their task is to decide whether a stimulus is a real word or a non‐word. Participants therefore must actively evaluate each stimulus and press different keys on a keyboard. This ensures engagement with the task and valid measurement of response times. Participants who do not accurately distinguish between words and non‐words are usually excluded from later analyses. Non‐words themselves serve only this methodological purpose and are not included in the later analyses.

**FIGURE 1:**
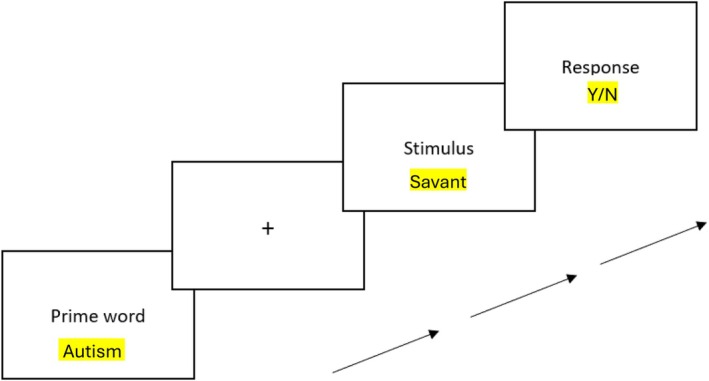
Procedure of each trial of the lexical decision task using an autism trial as an example.

In our study, participants were placed in front of a computer and instructed to indicate whether stimuli presented were existing German words or non‐words. Emphasis was placed on both the speed and accuracy of the decision. For each LDT, the respective SEN (autism, Down syndrome, dyslexia) was used as a prime word. Before the stimulus appeared, the prime word appeared for 300 ms followed by a fixation cross (see Figure 1).

To indicate whether it was a word or a non‐word, participants pressed a certain key: ‘y’ for yes and ‘n’ for no, assigned to the left and right index fingers. The stimulus disappeared as soon as the participants pressed one of the two keys. For example, after the prime word ‘autism’ and the fixation cross appeared, a participant had to decide whether ‘intelligent’ was a word or a non‐word and did so rightfully by pressing ‘y’ indicating it was a word. After a round of practice trials, participants proceeded to the actual experimental blocks, each consisting of 80 trials. Each block lasted about 5–7 min, and participants could take a break between the different LDTs. To avoid systematic order effects, the order of the three LDTs was randomized.

The actual stimuli were the traits used in the questionnaire to assess explicit stereotypes as well as matching German words that had just as many syllables and were just as common and non‐words that matched the syllable count of the used words. The matching German words were obtained using the SUBTLEX‐DE word frequencies (Brysbaert et al., 2011) to make sure that both the stereotypical and the non‐stereotypical words matched in terms of frequency. The procedure is illustrated in Figure 1.

#### Demographic data

To assess demographic information, we used a short questionnaire asking participants about age, gender, semester of study, type of teaching degree programme, subjects of study, and experience with autism, Down syndrome, and dyslexia.

### Data analysis

To answer subsections a and b of our research questions, we used one sample and paired *t*‐tests. Before analysing the implicit data, we computed error rates for each LDT and participant to exclude participants with an error rate above 15%. Trials with response times below 100 ms and above 10,000 ms were discarded. To answer subsection c of our research questions, we computed Kendall's tau as all variables demonstrated a non‐normal distribution. Following Rubin (2021), we applied an alpha correction because the analyses involved disjunction testing: Each hypothesis was evaluated using 10 tests, and the rejection of the joint hypothesis depended on at least one test being significant. Therefore, to control the familywise error rate at .05, we adjusted the alpha level to .005 per test.

## RESULTS

### Research question 1: Autistic pupils

#### Direct measures

Table 1 shows how the different stereotypical traits were rated for autistic pupils. To assess whether the ratings of each adjective significantly deviated from a neutral evaluation, we conducted one‐sample *t*‐tests comparing the ratings to the scale mean of 3.5. All traits were ranked significantly higher than the mean except for one.

**TABLE 1:** Rating of stereotypical traits regarding autism.

Traits	*M*	*SD*	Min	Max	*df*	*t*	*p*	*d*
Not socially competent^a^	**5.1**	.**93**	**2**	**6**	**75**	**14.73**	**<.0011**	**1.64**
Displaying behavioural problems^a^	**5.1**	.**95**	**3**	**6**	**75**	**14.24**	**<.001**	**1.63**
Savant	**4.9**	**1.2**	**1**	**6**	**75**	**10.07**	**<.001**	**1.16**
Impatient	**4.8**	**1.1**	**1**	**6**	**75**	**10.34**	**<.001**	**1.19**
Gifted	**4.8**	**1.2**	**2**	**6**	**75**	**9.21**	**<.001**	**1.06**
Uncommunicative	**4.7**	**1.2**	**1**	**6**	**75**	**8.15**	**<.001**	.**93**
Intelligent	**4.7**	**1.2**	**1**	**6**	**75**	**8.39**	**<.001**	.**96**
Impulsive	**4.5**	**1.3**	**1**	**6**	**75**	**6.67**	**<.001**	.**80**
Introverted	**4.2**	**1.5**	**1**	**6**	**75**	**4.24**	**<.001**	.**49**
Dependent	3.9	1.3	1	6	75	2.78	.007	.32

*Note*: All mean ratings of the traits that significantly differed from the mean of the scale (3.5) are marked in bold.

^a^ In German, the language in which this study was conducted, these are one‐worded adjectives.

#### Indirect measures

Regarding implicit stereotypical traits about autism, the mean error rate, meaning that participants falsely categorized the stimuli as words or non‐words, in the LDT was 7%. Three participants had to be excluded due to a high error rate. Seven trials (.01% of all trials) had to be discarded. To compare reactions to stereotypical and non‐stereotypical words, we computed a paired *t*‐test. On average, participants responded faster to stereotypical words (*M* = 1254.40 ms, *SD* = 675.46 ms) than non‐stereotypical words (*M* = 1739.55 ms, *SD* = 857.70 ms). The analysis revealed a statistically significant negative effect, with a medium effect size, *t*(72) = −3.97, *p* < .001; Cohen's *d* = −.64, 95% CI [−.97, −.32]. Table 2 shows the results for the *t‐*tests for each word individually. The pattern here is less clear: While some of the traits tapping low social skills showed significantly lower reaction times (indicating a stereotype), no clear picture emerged regarding the traits tapping high cognitive skills.

**TABLE 2:** Comparison of reaction time between stereotypical and non‐stereotypical words regarding autism.

Stereotypical word	Non‐stereotypical word	*t*	*df*	*p*	*d*
Not socially competent	Not unclear	**−3.33**	**70**	.**001**	.**38**
Displaying behavioural problems	Blasphemously	**−3.60**	**71**	**<.001**	.**41**
Savant	Mirror‐inverted	−.26	72	.795	.03
Impatient	Immediately	**−4.20**	**72**	**<.001**	.**48**
Gifted	Manicured	**−5.28**	**72**	**<.001**	.**61**
Uncommunicative	Exhibitionist	**−4.40**	**72**	**<.001**	.**50**
Intelligent	Held up	**−3.26**	**70**	.**001**	.**37**
Impulsive	Recovered	**−4.73**	**71**	**<.001**	.**39**
Introverted	Washed out	**−4.53**	**71**	**<.001**	.**53**
Dependent	Unfulfillable	.93	72	.353	.13

*Note*: Significant *t*‐tests are marked in bold.

#### Correlation between direct and indirect measures

Prior to conducting the correlation analysis, we tested all variables for normal distribution using the Shapiro–Wilk test. We then correlated the rating of the explicit trait, for example, ‘gifted’ for autism, and the relative reaction time towards the same word from the indirect measure. A positive relative reaction time would hereby indicate an implicit stereotypical trait. The results can be found in Table 3. Except for the trait ‘uncommunicative’, we found no significant correlations between explicit and implicit trait.

**TABLE 3:** Correlations between explicit and implicit stereotypical traits regarding autistic pupils.

Correlated explicit and implicit traits	Kendall's tau	*p*
Not socially competent	−.16	.081
Displaying behavioural problems	.08	.396
Savant	.03	.704
Impatient	−.05	.550
Gifted	−.14	.118
**Uncommunicative**	**−.20**	**<.005**
Intelligent	−.02	.741
Impulsive	.02	.860
Introverted	−.12	.178
Dependent	.05	.544

*Note*: Significant correlations are marked in bold.

### Research question 2: Pupils with Down syndrome

#### Direct measures

Table 4 shows how the different stereotypical traits were rated for pupils with Down syndrome analogous to Table 1. As expected, average ratings for the traits ‘warm‐hearted’, ‘tolerant’ and ‘good natured’ were substantially higher than the scale mean, with large effect sizes. Traits describing low intelligence (‘low achieving’, ‘stupid’) also showed average ratings above the scale mean, but the effect sizes were less pronounced.

**TABLE 4:** Rating of stereotypical traits regarding Down syndrome.

Traits	*M*	*SD*	Min	Max	*df*	*t*	*p*	*d*
Warm‐hearted	**5.1**	.**97**	**3**	**6**	**75**	**14.6**	**<.001**	**1.68**
Good‐natured	**5.0**	**1.2**	**1**	**6**	**75**	**10.85**	**<.001**	**1.24**
Dependent	**4.9**	**1**	**2**	**6**	**75**	**12.16**	**<.001**	**1.40**
Sincere	**4.8**	**1.3**	**1**	**6**	**75**	**8.79**	**<.001**	**1.01**
Displaying behavioural problems	**4.6**	**1.2**	**1**	**6**	**75**	**7.71**	**<.001**	.**88**
Awkward	**4.5**	**1.2**	**1**	**6**	**75**	**7.13**	**<.001**	.**82**
Impatient	**4.3**	**1.2**	**2**	**6**	**75**	**6.03**	**<.001**	.**70**
Low achieving	**4.3**	**1.6**	**1**	**6**	**75**	**4.70**	**<.001**	.**54**
Tolerant	**4.3**	**1.4**	**1**	**6**	**75**	**4.93**	**<.001**	.**57**
Stupid	**4.1**	**1.4**	**1**	**6**	**75**	**3.58**	**<.001**	.**41**

*Note*: All mean ratings of the traits that significantly differed from the mean of the scale (3.5) are marked in bold.

#### Indirect measures

Regarding Down syndrome, the mean error rate was 4.05%. One participant had to be excluded due to a high error rate. Five trials (<.01%) had to be discarded. On average, participants responded faster to stereotypical words (*M* = 1101.80 ms, *SD* = 513.24 ms) than non‐stereotypical words (*M* = 1486.07 ms, *SD* = 806.64 ms). The paired *t*‐test revealed a statistically significant negative effect, with a medium effect size, *t*(74) = −3.48, *p* < .001; Cohen's *d* = −.57, 95% CI [−.60, −.17]. Table 5 shows the results for the *t‐*tests for each word individually. Overall, significantly lower reaction times only emerged for five out of the 10 stereotypical traits, with small effect sizes and no clear pattern.

**TABLE 5:** Comparison of reaction time between stereotypical and non‐stereotypical words regarding Down syndrome.

Stereotypical word	Non‐stereotypical word	*t*	*df*	*p*	*d*
Warm‐hearted	Moved up	**−4.71**	**74**	**<.001**	.**51**
Good‐natured	Fossilized	−1.74	74	.090	.20
Dependent	Unfulfillable	1.12	74	.266	.13
Sincere	Surrounded	**−2.97**	**74**	.**004**	.**34**
Displaying behavioural problems	Blasphemously	**−4.94**	**74**	**<.001**	.**57**
Awkward	Contaminated	**−5.45**	**73**	**<.001**	.**47**
Impatient	Immediately	**−4.11**	**73**	**<.001**	.**36**
Low achieving	Reverently	**−5.16**	**72**	**<.001**	.**24**
Tolerant	Solemnly	−.95	73	.344	.16
Stupid	New	−1.50	74	.137	.17

*Note*: Significant *t*‐tests are marked in bold.

#### Correlation between explicit and implicit associations

As can be seen in Table 6, the explicit and implicit traits did not correlate with each other.

**TABLE 6:** Correlations between explicit and implicit stereotypical traits regarding pupils with Down syndrome.

Correlated explicit and implicit stereotypical traits	Kendall's tau	*p*
Warm‐hearted	.07	.406
Good‐natured	−.08	.353
Dependent	.10	.249
Sincere	−.02	.833
Displaying behavioural problems	−.07	.427
Awkward	−.01	.896
Impatient	−.04	.641
Low achieving	−.10	.251
Tolerant	.01	.946
Stupid	−.10	.260

### Research question 3: Pupils with dyslexia

#### Direct measures

Table 7 shows how the different stereotypical traits were rated for pupils with dyslexia analogous to Tables 1 and 4. As expected, the traits ‘low‐achieving’, ‘stupid’, and ‘lazy’ were rated particularly highly; however, there were also several other traits strongly attributed to dyslexic students, such as ‘impatient’, ‘awkward’, ‘warm‐hearted’, and ‘dependent’, while traits pertaining to social problems were not above the scale mean. Overall, the effect sizes were lower compared with the other two student groups, indicating less salient explicit stereotypical traits regarding dyslexic students.

**TABLE 7:** Rating of stereotypical traits regarding dyslexia.

Traits	*M*	*SD*	Min	Max	*df*	*t*	*p*	*d*
Low achieving	**4.6**	**1.5**	**1**	**6**	**75**	**6.41**	**<.001**	.**74**
Impatient	**4.3**	**1.1**	**2**	**6**	**75**	**6.44**	**<.001**	.**74**
Lazy	**4.3**	**1.5**	**1**	**6**	**75**	**4.68**	**<.001**	.**54**
Awkward	**4.2**	**1.2**	**1**	**6**	**75**	**4.89**	**<.001**	.**56**
Stupid	4	1.6	1	6	75	2.91	.005	.33
Warm‐hearted	3.9	1.2	1	6	75	2.88	.005	.33
Dependent	3.9	1.2	1	6	75	2.83	.006	.32
Displaying behavioural problems	3.4	1.5	1	6	75	−.54	.594	−.06
Introverted	3.3	1.2	1	6	75	−1.59	.116	−.18
Uncommunicative	3.2	1.1	1	6	75	−2.50	.015	−.29

*Note*: All mean ratings of the traits that significantly differed from the mean of the scale (3.5) are marked in bold.

#### Indirect measures

Regarding dyslexia, the mean error rate was 3.52%. No participant had to be excluded due to a high error rate. Six trials (<.01%) had to be discarded. On average, participants responded faster to stereotypical words (*M* = 1164.79 ms, *SD* = 550.01 ms) than non‐stereotypical words (*M* = 1664.03 ms, *SD* = 883.07 ms). The analysis revealed a statistically significant negative effect, with a medium effect size, *t*(75) = −4.18, *p* < .001; Cohen's *d* = −.68, 95% CI [−1.01, −.35]. Table 8 shows the results for the *t‐*tests for each word individually. For eight out of 10 stereotypical words, substantially lower reaction times were observed. Interestingly, the traits indicating social problems that had not been rated particularly highly in the explicit measure now showed significantly lower reaction times, indicating shared stereotypical traits.

**TABLE 8:** Comparison of reaction time between stereotypical and non‐stereotypical words regarding dyslexia.

Stereotypical word	Non‐stereotypical word	*t*	*df*	*p*	*d*
Low achieving	Reverently	**−4.53**	**74**	**<.001**	.**40**
Impatient	Immediately	−2.54	75	.013	.30
Lazy	Smooth	**−3.43**	**75**	**<.001**	.**40**
Awkward	Contaminated	**−3.62**	**74**	**<.001**	.**42**
Stupid	New	−1.02	75	.309	.12
Warm‐hearted	Moved up	**−4.18**	**74**	**<.001**	.**48**
Dependent	Unfulfillable	−.05	74	.960	.06
Displaying behavioural problems	Blasphemously	**−3.70**	**75**	**<.001**	.**42**
Introverted	Washed out	**−5.26**	**75**	**<.001**	.**60**
Uncommunicative	Exhibitionist	**−4.03**	**73**	**<.001**	.**48**

*Note*: Significant *t*‐tests are marked in bold.

#### Correlation between explicit and implicit associations

Table 9 shows Kendall's rank correlations for all traits. Again, there were no significant correlations between the explicit and implicit traits.

**TABLE 9:** Correlations between explicit and implicit stereotypical traits regarding pupils with dyslexia.

Correlated explicit and implicit associations	Kendall's tau	*p*
Low achieving	−.03	.745
Impatient	−.09	.299
Lazy	.09	.269
Awkward	.04	.623
Stupid	−.09	.268
Warm‐hearted	.03	.627
Dependent	.06	.454
Displaying behavioural problems	.02	.805
Introverted	−.06	.491
Uncommunicative	−.09	.324

## DISCUSSION

The aim of the study was to investigate stereotypical traits that pre‐service teachers associate with pupils with different SEN and explore the relationship between them. Regarding explicit stereotypical traits, results showed that pre‐service teachers associated a number of traits with autistic pupils, pupils with Down syndrome, and, to a lower degree, pupils with dyslexia. This overall pattern mirrors the findings of Schell et al. (2024), the only other study known to the authors investigating similar traits for all three groups.

As for autism, a closer comparison showed that positive traits like being ‘gifted’ and ‘intelligent’ were attributed to autistic pupils to a similar degree in both studies, suggesting some stability in the perception of cognitive abilities. This is also in line with prior research on the general public suggesting all autistic people are seen as gifted or very intelligent while lacking social skills (Draaisma, 2009). Being a ‘savant’ and ‘introverted’ was rated lower in the present study. On the contrary, negative traits such as ‘displaying behavioural problems’ and being ‘impulsive’ received higher ratings in the present study. This points towards a slightly stronger endorsement of socially negative stereotypes in the present sample.

Regarding Down syndrome, there were many similarities in terms of ratings between both studies. However, similar to autism, some positive traits such as being ‘warm‐hearted’ and ‘good‐natured’ were rated slightly lower in our sample while ‘displaying behavioural problems’ was rated higher. This, again, may indicate slightly more negative salient or readily endorsed stereotypes; though, overall, the picture did not differ much. The positive traits associated with pupils with Down syndrome both in Schell et al.'s (2024) study and the current one are in line with findings of how non‐professionals view people with Down syndrome (Gilmore et al., 2003). However, Gilmore et al. (2003) reported that teachers endorsed more positive traits and fewer negative ones, which cannot be said for our study or Schell et al. (2024).

Finally, as for dyslexia, both studies showed less pronounced stereotypes compared with autism and Down syndrome with few traits receiving consistently high ratings (Schell et al., 2024). Notably, ‘low achieving’, ‘stupid’ and ‘lazy’ seemed to be strongly endorsed in both studies, while ‘lazy’ was rated even higher in our study. The findings mirror widespread stereotypes held by individuals outside the educational context (Haft et al., 2022; Piotrowska & Barratt, 2024).

While some of the stereotypical characteristics may apply to some of these pupils, issues might arise when these stereotypes prevent (future) teachers from recognizing the different and unique characteristics of each pupil. The interpretation of these results in terms of social desirability requires caution: We expected pre‐service teachers to be more hesitant to rate these groups in such a stereotypical way due to social norms. Compared with other disadvantaged groups in the school context, for example, ethnic minority students, the explicit reporting of negative stereotypical traits is unusual: Research on explicit stereotypes regarding ethnic minority students shows mainly positive associations due to social desirability and negative stereotypes being seen as not socially acceptable (Glock et al., 2020). Our findings, however, may suggest that pre‐service teachers perceive it as socially acceptable to report negative stereotypes about certain groups of pupils with SEN, although a general conclusion concerning the role of social desirability is difficult and multiple mechanisms may be involved. One explanation could be that the diagnosis and the traditional medical understanding that goes with it might lead (pre‐service) teachers to see the stereotypical characteristics as part of the diagnosis. In a book section on social perception, Jussim (2005) argues that these beliefs could reflect base‐rate knowledge that can be accurate on a group level but still requires cautious application on an individual level. For example, reporting that all autistic pupils are ‘uncommunicative’ may be perceived as factual and professionally acceptable based on the autism diagnosis. For ethnic minority students, such an attribution cannot be made, as it is solely demographic information. Previous research on a possible relation between stereotypes towards students with SEN and social desirability finds mixed effects (Hunt, 2006; Kim et al., 2015).

However, it is important to note that this attribution‐to‐diagnosis explanation cannot apply to all stereotypical traits in this study. Some, for example, the idea that all pupils with Down syndrome or dyslexia are ‘stupid’ or ‘lazy’, reflect harmful judgements rather than clinically descriptive characteristics. Another possible explanation, therefore, goes beyond the medical understanding and focuses on what is supposedly accepted in a society and what is not. A recent study by Ayala et al. (2024) found that teachers openly reported agreement with negative stereotypical traits concerning the learning abilities of Haitian pupils in Chile as well as negative implicit bias. The authors argue that this challenges the traditional expectation of social desirability bias and instead suggest that teachers do not adjust their answers if the answers are socially acceptable in the context. They note that Haitians are an extremely marginalized group in Chile, much more than many other groups (Ayala et al., 2024). In other words, teachers may think there are real differences in academic skills or behaviour because of the societal view on this particular group of people, not seeing this as discriminatory. This reasoning supports the idea that explicit endorsement of negative stereotypical traits in our study may likewise reflect socially legitimized beliefs, particularly when these stereotypes are tied to diagnostic labels rather than demographic characteristics.

Interpreting the results from the LDTs, in line with research on indirect methods and other marginalized groups (Denessen et al., 2022), pre‐service teachers seemed to also hold implicit stereotypes towards autistic pupils, pupils with Down syndrome, and pupils with dyslexia. Implicit associations consistent with common stereotypes were present for all three groups of pupils. In contrast to the findings regarding explicit stereotypical traits, these general results seemed less surprising. On closer inspection, it was somewhat surprising which implicit stereotypical traits were strongly associated, especially in comparison to the explicitly queried ones.

As for autistic pupils, almost all traits that were explicitly rated high also seemed to be strongly implicitly associated with autism, except for being ‘savant’ and ‘dependent’. These two traits may be related in an interesting way: A savant is usually someone with an exceptional skill in one specific area, but who often has difficulties in other parts of life (Treffert, 2009, 2014). In contrast, when someone is described as intelligent, it is generally assumed they are competent across a wider range of tasks and more independent overall (Gottfredson, 1997). Thus, even though a savant might show extraordinary talent, they might also be seen as more dependent in everyday or social situations.

Regarding Down syndrome, the overall picture was also similar for explicit and implicit stereotypical traits. Yet, it was surprising that the implicit associations did not include the positive traits of being good‐natured and tolerant as well as the negative traits of being dependent and stupid. It therefore might be useful for future research to explore these implicit stereotypes through an exploratory approach to gain a better understanding.

For pupils with dyslexia, being ‘low achieving’, ‘lazy’ and ‘awkward’ were reported and associated both explicitly and implicitly. As opposed to the other two groups, more traits were associated only implicitly: being ‘warm‐hearted’, ‘introverted’ and ‘uncommunicative’ as well as ‘displaying behavioural problems’. While the core stereotype remained the same, implicit associations revealed that pre‐service teachers may not actually be less biased towards pupils with dyslexia but in this case might feel some level of pressure to not explicitly express further negative or paternalistic traits. Consequently, the view on pupils with dyslexia seems to differ from the other two groups.

Regarding the relationship between explicit and implicit stereotypes, the authors initially expected low or even no correlations due to the possible influence of social desirability and the fact that they tap into different cognitive processes. It was, however, surprising that for all three groups, with one exception, explicit and implicit traits did not correlate at all. In the literature, most research is focused on the IAT (Hofmann et al., 2005). They attribute the differences in correlations between explicit measures and the IAT to certain factors such as the conceptual alignment of the measures used, methodological factors, and the level of spontaneity of the explicit self‐reports (Hofmann et al., 2005). The authors assume that their findings also apply for other indirect measures, though to the bset of our knowledge, this has never been empirically investigated. As for our study, the conceptual alignment should be high as we used the exact same traits to measure both explicit and implicit stereotypes. Regarding spontaneity, it is likely that the pre‐service teachers thought about the explicit stereotypes more deliberately compared with the implicit stereotypes. A possible explanation could also be that the relationship between explicit and implicit stereotypes varied individually between the pre‐service teachers. This explanation is supported by the relatively large standard deviations; hence, the variance of the data around the mean for both the ratings and the reaction times.

### Limitations and future research

There are several possible limitations of this study. First, our sample was rather small and locally restricted, which limits its generalisability. Furthermore, we did not consider other attributes of the three groups of pupils like gender, ethnicity, or socio‐economic status. From an intersectional perspective, it is thinkable that these attributes and the stereotypes associated with them could interact with the stereotypes associated with autism, Down syndrome, or dyslexia. For example, there is a discussion about gender differences in autism (Lai et al., 2015), an interplay of two attributes that could potentially play a role when it comes to (pre‐service) teachers' stereotypes. Future research could therefore expand this research by looking at such interplays.

Regarding SEN, we did not investigate all groups that fall under this umbrella term; we did not cover all groups within the neurodivergent spectrum. Our study is therefore only a first step into a complex topic, and it would be interesting to expand this research to other groups, such as pupils with ADHD or gifted pupils.

As for the design of the questionnaire used in the study, we did not include neutral or counter‐stereotypical traits. While this approach was consistent with our aim to examine how pre‐service teachers rate specific stereotypical traits, it limits the ability to detect counter‐stereotypical associations across the full spectrum of possible traits. Future research could therefore include reversed or neutral items. In addition, our rationale for selection was to select items that (a) reflect the traits most frequently discussed in the literature for the respective groups and (b) showed high salience in previous studies, including Schell et al. (2024). In this sense, the list should be seen as representative rather than exhaustive, and there might be additional adjectives that are associated with the respective groups.

As for the lexical decision tasks, we chose the matching words based on the SUBTLEX‐DE word frequencies (Brysbaert et al., 2011) because studies showed that it outperformed other databases when it came to LDT use (Brysbaert et al., 2011; Heister & Kliegl, 2012). However, being published in 2011, it is possible that the frequencies of words may have shifted slightly since the database was compiled, which may have compromised the comparability of some of the word matches. A recent study (Haeuser & Kray, 2025) indeed questions the generalizability of SUBTLEX‐DE to contextual reading paradigms; however, LDTs seem to highlight exactly the kinds of whole‐word recognition processes that databases like SUBTLEX‐DE capture well. Given that our study employed lexical decision, SUBTLEX‐DE remains an appropriate and validated frequency measure for our purposes.

A further limitation concerns the use of non‐stereotypical words in the LDTs. Ideally, all words would have described personal traits just like the stereotypical words. However, given the need to match lexical properties such as word length and frequency, the available pool of suitable words was limited. While this introduces the possibility that differences in response times could partly reflect variations in personal relevance, it is important to note that in our data both personal and non‐personal non‐stereotypical words differed significantly from the stereotypical ones.

### Theoretical and practical implications

We found explicit and implicit stereotypes regarding different groups of pupils with SEN. First of all, the finding that these stereotypes are held by (pre‐service) teachers, not only by the general public as sometimes shown in other studies (Canton et al., 2023; Draaisma, 2009), is especially concerning. Teachers are key figures in shaping the school environment, and their beliefs can influence classroom expectations, support behaviour, and pupils' self‐perceptions. This is often called the Pygmalion or Rosenthal effect (Rosenthal & Fode, 1963; Rosenthal & Jacobson, 1968). The overlap in stereotypes suggests that even professional training at university may not sufficiently challenge pervasive social narratives.

In the school context, the two forms of stereotypes – explicit and implicit stereotypes – could play a role in different situations: Following Fazio's (1995) model, explicit stereotypes may be more influential in situations where (pre‐service) teachers' high motivation and/or high cognitive capacity allows for controlled behaviour. In contrast, implicit stereotypes may influence automatic behaviour in stressful situations, such as busy classroom situations, where (pre‐service) teachers have little time to think. Our findings may help identify which types of situations are particularly relevant for the different groups of pupils. For both autism and Down syndrome, explicit and implicit stereotypical associations appear to be similarly pronounced, suggesting that both controlled and automatic responses may be influential. For dyslexia, however, implicit stereotypes were more prominent, which could indicate that unaware, automatic behaviour may play a larger role, especially in high‐pressure school contexts. Further research is needed to investigate these possible influences on behaviour.

As the stereotypes differ depending on the group, consequently the behaviour of (pre‐service) teachers might differ too. For example, Cuddy et al. (2007) combined stereotypes based on the Stereotype Content Model (Fiske et al., 2002) with specific emotional reactions and behavioural tendencies. While we did not assess the dimensions Fiske et al. (2002) used and can therefore not apply Cuddy et al.'s (2007) BIAS map, parallels to our work can still be drawn. For instance, different traits associated with the groups of pupils might lead to different forms of engagement, support, or avoidance. Certain profiles may evoke overprotective or paternalistic responses, while others might lead to social distancing or reduced instructional effort. Future research should therefore investigate whether these traits do indeed lead to different behaviour in the classroom and which traits in particular are important predictors of behaviour.

Given that pre‐service teachers on the one hand seem to share a lot of stereotypical ideas that the general public has about autistic pupils, pupils with Down syndrome and pupils with dyslexia, and that research on stereotypes and teachers' beliefs suggests that if not challenged, these stereotypes could influence their behaviour as future teachers in a biased way, pre‐service teachers should be made aware of their stereotypes in general, as well as of the effects of both explicit and implicit stereotypes specifically. It is important to recognize the strengths and weaknesses of children with SEN, but not based on an unreflected stereotypical assessment, but on an individual diagnosis. Therefore, both knowledge about the influence of biases on (everyday) diagnostic decision in the classroom as well as knowledge about these groups would be a useful addition to teacher training.

In conclusion, the study showed that pre‐service teachers have both explicit as well as implicit stereotypes towards autistic pupils, pupils with Down syndrome, and pupils with dyslexia. The strong stereotype content found may reveal a potential problem for successful inclusion in two ways: On the one hand, implicit stereotypes might influence automatic behaviour and lead to teachers overlooking the actual characteristics and needs of individual pupils. On the other hand, the strong explicit stereotypes could not only influence controlled behaviour but could also be an indicator that pre‐service teachers do not find it problematic or socially undesirable to have such stereotypes. At the same time, the found stereotype content differed between groups and could be an indicator of how differently (pre‐service) teachers treat these groups.

## AUTHOR CONTRIBUTIONS

**Charlotte S. Schell:** Conceptualization; project administration; methodology; data curation; formal analysis; investigation; writing – original draft; visualization. **Hannah Kleen:** Conceptualization; writing – review and editing; supervision. **Charlotte Dignath:** Conceptualization; writing – review and editing; supervision; funding acquisition. **Nathalie John:** Writing – review and editing; conceptualization. **Mareike Kunter:** Conceptualization; writing – review and editing; supervision; funding acquisition.

## CONFLICT OF INTEREST STATEMENT

The authors declare no conflicts of interest.

## Supporting information


Data S1.

## Data Availability

The data that support the findings of this study are available from the corresponding author upon reasonable request.

## Footnotes

^1^
In this article, we are using both identity‐first (‘autistic pupils’) as well as person‐first (pupils with Down syndrome, pupils with dyslexia') language in order to adapt to the different preferences of the respective groups.
