# Supplementary material for: Pre‐service teachers' explicit and implicit stereotypes towards pupils with different special educational needs
Source: Br J Educ Psychol. 2025 Oct 7;96(2):612–30. doi: 10.1111/bjep.70041 (PMC13155035; doi:10.1111/bjep.70041)
Supplement: Supplementary file 1 — Data S1. [file BJEP-96-612-s001.docx]

**Supplementary Material**

**Supplementary Material A – Stimuli**

**Table A1**

*Original German stereotypical and non-stereotypical words used in the Lexical Decision Task for autism*

| Stereotypical Word | Non-Stereotypical Word |
| --- | --- |
| nicht sozialkompetent | nicht unübersichtlich |
| unkommunikativ | exhibitionistisch |
| inselbegabt | spiegelverkehrt |
| ungeduldig | unverzüglich |
| hochbegabt | manikürt |
| intelligent | aufgehalten |
| verhaltensauffällig | gotteslästerlich |
| introvertiert | ausgewaschen |
| unselbstständig | unerfüllbar |
| impulsiv | genesen |

**Table A2**

*Non-words used in the Lexical Decision Task for autism*

| Non-words |
| --- |
| minapalutiv |
| birotungsrisistant |
| sansitionell |
| überflassig |
| gerimmert |
| kuttersaalenallein |
| gestronomisch |
| unterhiltsam |
| abnarmil |
| internotionell |
| kultitaskingfahig |
| ungehauer |
| wohlbekütet |
| zagellos |
| eigenhundig |
| aufsahenerragend |
| fakettenreich |
| unwillkarlich |
| deuerheft |

**Table A3**

*Original German stereotypical and non-stereotypical words used in the Lexical Decision Task for Down syndrome*

| Stereotypical Word | Non-Stereotypical Word |
| --- | --- |
| warmherzig | vorverlegt |
| gutmütig | versteinert |
| unselbstständig | unerfüllbar |
| aufrichtig | umgeben |
| unbeholfen | kontaminiert |
| ungeduldig | unverzüglich |
| leistungsschwach | ehrfurchtsvoll |
| verhaltensauffällig | gotteslästerlich |
| tolerant | feierlich |
| dumm | neu |

**Table A4**

*Non-words used in the Lexical Decision Task for Down syndrome*

| Non-words |
| --- |
| abnarmil |
| gerimmert |
| unterhiltsam |
| ausgefefft |
| gestronomisch |
| abwachslungsreich |
| ehrgozig |
| kuttersaalenallein |
| jumorvoll |
| brin |
| deuerheft |
| zagellos |
| unwillkarlich |
| körferflich |
| fakettenreich |
| eigenhundig |
| gemunsam |
| aufsahenerragend |
| intrihant |

**Materials for dyslexia**

**Table A5**

*Original German stereotypical and non-stereotypical words used in the Lexical Decision Task for dyslexia*

| Stereotypical Word | Non-Stereotypical Word |
| --- | --- |
| leistungsschwach | ehrfurchtsvoll |
| ungeduldig | unverzüglich |
| unselbstständig | unerfüllbar |
| dumm | neu |
| faul | glatt |
| unbeholfen | kontaminiert |
| warmherzig | vorverlegt |
| verhaltensauffällig | gotteslästerlich |
| unkommunikativ | exhibitionistisch |
| introvertiert | ausgewaschen |

**Table A6**

*Non-words used in the Lexical Decision Task for dyslexia*

| Non-words |
| --- |
| jumorvoll |
| unterhiltsam |
| gestronomisch |
| brin |
| jund |
| abwachslungsreich |
| ausgefefft |
| kuttersaalenallein |
| birotungsrisistant |
| sansitionell |
| intrihant |
| unwillkarlich |
| fakettenreich |
| vuel |
| kut |
| eigenhundig |
| körferflich |
| aufsahenerragend |
| kultitaskingfahig |

**Supplementary Material B – Mean Response Latencies**

**Table B1**

*Mean response latencies and standard deviations for stereotypical and non-stereotypical words regarding autism in seconds*

| Stereotypical word | *M* | *SD* | Non-stereotypical word | *M* | *SD* |
| --- | --- | --- | --- | --- | --- |
| Not Socially Competent | 1.928 | 1.215 | Not unclear | 2.813 | 0.692 |
| Displaying Behavioural Problems | 1.334 | 0.876 | Blasphemously | 1.855 | 0.874 |
| Savant | 1.436 | 1.195 | Mirror-inverted | 1.476 | 0.817 |
| Impatient | 0.895 | 0.356 | Immediately | 1.412 | 1.071 |
| Gifted | 0.937 | 0.419 | Manicured | 1.713 | 1.272 |
| Uncommunicative | 1.473 | 1.189 | Exhibitionist | 2.319 | 1.181 |
| Intelligent | 1.049 | 0.678 | Held up | 1.546 | 1.281 |
| Impulsive | 0.884 | 0.376 | Recovered | 1.291 | 0.753 |
| Introverted | 1.135 | 0.976 | Washed out | 1.638 | 0.846 |
| Dependent | 1.470 | 1.031 | Unfulfillable | 1.329 | 0.877 |

**Table B2**

*Mean response latencies and standard deviations for stereotypical and non-stereotypical words regarding Down syndrome in milliseconds*

| Stereotypical word | *M* | *SD* | Non-stereotypical word | *M* | *SD* |
| --- | --- | --- | --- | --- | --- |
| Warm-hearted | 0.984 | 0.630 | Moved up | 1.499 | 0.973 |
| Good-natured | 1.149 | 0.826 | Fossilised | 1.441 | 1.140 |
| Dependent | 1.494 | 1.003 | Unfulfillable | 1.359 | 1.058 |
| Sincere | 1.063 | 0.702 | Surrounded | 1.447 | 1.051 |
| Displaying Behavioural Problems | 1.250 | 0.662 | Blasphemously | 2.048 | 1.447 |
| Awkward | 0.994 | 0.452 | Contaminated | 1.582 | 0.990 |
| Impatient | 0.974 | 0.634 | Immediately | 1.356 | 0.954 |
| Low achieving | 1.099 | 0.615 | Reverently | 1.803 | 1.146 |
| Tolerant | 1.100 | 0.646 | Solemnly | 1.234 | 0.870 |
| Stupid | 0.888 | 0.557 | New | 1.044 | 0.833 |

**Table B3**

*Mean response latencies and standard deviations for stereotypical and non-stereotypical words regarding dyslexia in milliseconds*

| Stereotypical word | *M* | *SD* | Non-stereotypical word | *M* | *SD* |
| --- | --- | --- | --- | --- | --- |
| Low achieving | 1.171 | 0.572 | Reverently | 1.924 | 1.062 |
| Impatient | 0.971 | 0.529 | Immediately | 1.343 | 1.049 |
| Lazy | 0.847 | 0.317 | Smooth | 1.197 | 0.892 |
| Awkward | 1.071 | 0.692 | Contaminated | 1.531 | 1.144 |
| Stupid | 0.876 | 0.482 | New | 0.961 | 0.696 |
| Warm-hearted | 0.977 | 0.563 | Moved up | 1.679 | 1.381 |
| Dependent | 1.584 | 1.093 | Unfulfillable | 1.597 | 1.187 |
| Displaying Behavioural Problems | 1.463 | 1.045 | Blasphemously | 2.198 | 1.071 |
| Introverted | 1.113 | 0.647 | Washed out | 1.782 | 1.100 |
| Uncommunicative | 1.551 | 1.145 | Exhibitionist | 2.397 | 1.053 |
